# A review of indoor air quality interventions and child neurobehavioral outcomes

**DOI:** 10.1038/s41390-026-05401-w

**Published:** 2026-08-27

**Authors:** Aimée Vester, Patrick H. Ryan, Kimberly Yolton, Jareen K. Meinzen-Derr, Nicholas C. Newman

**Affiliations:** 1 Division of General & Community Pediatrics, Cincinnati Children’s Hospital Medical Center, Cincinnati, OH USA; 2 Department of Pediatrics, University of Cincinnati College of Medicine, Cincinnati, OH USA; 3 Division of Biostatistics and Epidemiology, Cincinnati Children’s Hospital Medical Center, Cincinnati, OH USA; 4 Department of Environmental and Public Health Sciences, University of Cincinnati College of Medicine, Cincinnati, OH USA; 5 Center for Clinical and Translational Science and Training, Cincinnati Children’s Hospital Medical Center, Cincinnati, OH USA; 6 Department of Otolaryngology, University of Cincinnati College of Medicine, Cincinnati, OH USA

## Abstract

**Abstract:**

Children are uniquely vulnerable to the neurotoxic effects of air pollution because their brains continue to develop throughout childhood. Indoor air pollution comprises a mixture of several pollutants and is influenced by both indoor and outdoor air pollutant sources. Growing evidence from animal models and epidemiological studies illustrates the adverse effects of air pollutants, especially particulate matter ≤2.5 μm (PM_2.5_), on cognitive function and academic performance due to its ability to generate oxidative stress and elicit neuroinflammation. Interventions such as air filtration can meaningfully reduce indoor concentrations of pollutants such as PM_2.5_. Given the significant time children spend indoors in home, school, and childcare settings, indoor air quality interventions are apotential tool to mitigate adverse health effects of indoor air pollutants in children. However, most interventional studies of indoor air pollution focus on improving respiratory outcomes, such as asthma. Here, we conducted a narrative review to collate studies on indoor air pollution interventions that assess neurobehavioral outcomes. Ultimately, our goal was to highlight potential future directions for additional interventional studies that may contribute to public health efforts to optimize neurobehavioral outcomes in children.

**Impact:**

- Mounting evidence suggests indoor air pollution presents a public health concern for neurobehavioral outcomes.
- Children are particularly vulnerable to the adverse effects of indoor air pollution and subsequent neurobehavioral outcomes.
- While studies illustrate potential benefits of indoor air pollution interventions on respiratory outcomes, less is known about neurobehavioral outcomes.
- This narrative review adds to existing literature by summarizing studies of indoor air pollution interventions and neurobehavioral outcomes published in the past 20 years.
- This review discusses potential next steps for future interventional studies on indoor air quality and child neurobehavior.

## Introduction

While the United States (U.S.) Environmental Protection Agency (EPA) regulates outdoor air pollution, it does not currently regulate indoor air quality in homes, schools, or childcare settings. American children spend, on average, 80% of their time indoors at home, at school, or in childcare settings.^1^ Children are particularly vulnerable to the effects of air pollution due to their relatively high minute ventilation (i.e., the volume of air inhaled and exhaled in 1 min), rapidly developing organs, and immature immune and detoxification systems.^2^ Initial studies of ambient air pollution and child health focused on its impact on the respiratory and immune systems, particularly allergies and asthma.^3–9^ However, the developing nervous system is also vulnerable to environmental exposures, and there is increasing evidence from toxicologic and epidemiologic research that early-life exposure to air pollution adversely affects neurobehavioral outcomes.^10^ While much of the research on air pollution and neurobehavioral outcomes has focused on outdoor air pollution, several component pollutants of outdoor air pollution associated with adverse health outcomes are also found in the indoor environment including particulate matter, nitrogen dioxide (NO_2_) carbon dioxide (CO_2_), and volatile organic compounds (VOCs). Indoor air quality is influenced by many factors, including amounts of outdoor air pollution that penetrate the building, how much and the type of pollution that is generated indoors, and how well that indoor air pollution is diluted or filtered out of the air.^11^

The largest body of evidence relating air pollutants to neurobehavioral outcomes is available for particulate matter, especially particulate matter <2.5 μm (PM_2.5_). Particulate matter can arise from natural or anthropogenic sources, such as traffic-related air pollution, and comprises several different chemicals of different aerodynamic diameters. PM_2.5_ can translocate to the brain and elicit neuroinflammation, because of its size, and can also induce systemic inflammation and oxidative stress that may impact neurobehavior.^12^ Recent meta-analyses illustrate that prenatal and early-life exposure to PM_2.5_ is associated with neurobehavioral outcomes, including increased risk of autism spectrum disorder^13,14^ and adverse cognitive outcomes such as decreased intelligence quotient (IQ).^15,16^ Evidence on associations between PM_2.5_ and attention-deficit hyperactivity disorder (ADHD) is also suggestive but less consistent.^17–19^

Although the precise mechanisms of neurological injury due to these air pollutants are not known, current evidence suggests that pollutant-induced inflammation and oxidative stress are involved.^20^ For example, Brunst et al. evaluated 145 adolescents and examined exposure to traffic-related air pollution over the preceding 12 months. They noted that high levels of traffic-related air pollution were associated with increased brain myo-inositol concentrations, a metabolite implicated in neuroinflammation, via magnetic resonance spectroscopy.^17^ Another analysis from the same cohort by Beckwith et al. observed reduced cortical thickness via brain magnetic resonance imaging in the high versus low traffic-related air pollution groups,^21,22^ suggesting that air pollution may also impact brain structure.

In addition to PM_2.5_, epidemiologic studies also correlate early-life exposures to volatile organic compound (VOC) mixtures with developmental, motor and, communication delays;^23^ polycyclic aromatic hydrocarbons (PAHs) with increased risk of social behavior, attention, and motor delays;^12,24^ and nitrogen oxide with ADHD.^25^

Given that air pollution is associated with adverse health outcomes and the importance of the indoor environment for children, indoor air filtration directed at reducing PM_2.5_ by utilizing tools such as High Efficiency Particulate Air (HEPA) filters, has been proposed as a potential strategy to improve health outcomes.^26–28^ A recent systematic review of indoor air quality interventions for children with asthma concluded that air filtration shows early promise for improving respiratory symptoms.^29^ Although numerous studies of indoor air pollution interventions have measured respiratory health outcomes,^26,30–44^ fewer have measured neurobehavioral outcomes. Thus, our objective in this narrative review was to synthesize existing literature on indoor air pollution interventions designed to measure a neurobehavioral outcome and provide guidance on future research directions.

## Methods

To identify relevant research articles, we queried PubMed, Scopus, and Embase databases. The initial search, completed on September 22, 2025, focused on neurodevelopment, cognition, and mental health as key outcomes. A follow-up search on September 30, 2025, broadened the scope to include child outcomes beyond neurobehavioral measures, ensuring we captured relevant outcomes such as academic achievement. To ensure that recent publications were not missed, we repeated this same literature search again on March 10, 2026. Search strategies were developed by our institutional librarian (Supplemental Table S1) and incorporated keywords such as “indoor air pollution”, “air quality”, “indoor filtration”, “PM2.5 particulate matter”, “HEPA filtration”, and “health outcome”. In the first round of screening, key search terms for outcomes included “neurodevelopment”, “cognition”, and “mental health.” However, we broadened our search terms in the second round to ensure all possible outcomes related to neurodevelopment were included. We switched our key search terms for outcomes to “health condition”, “disorder”, and “outcome.” This approach primarily added articles examining academic outcomes.

**Fig. 1:**
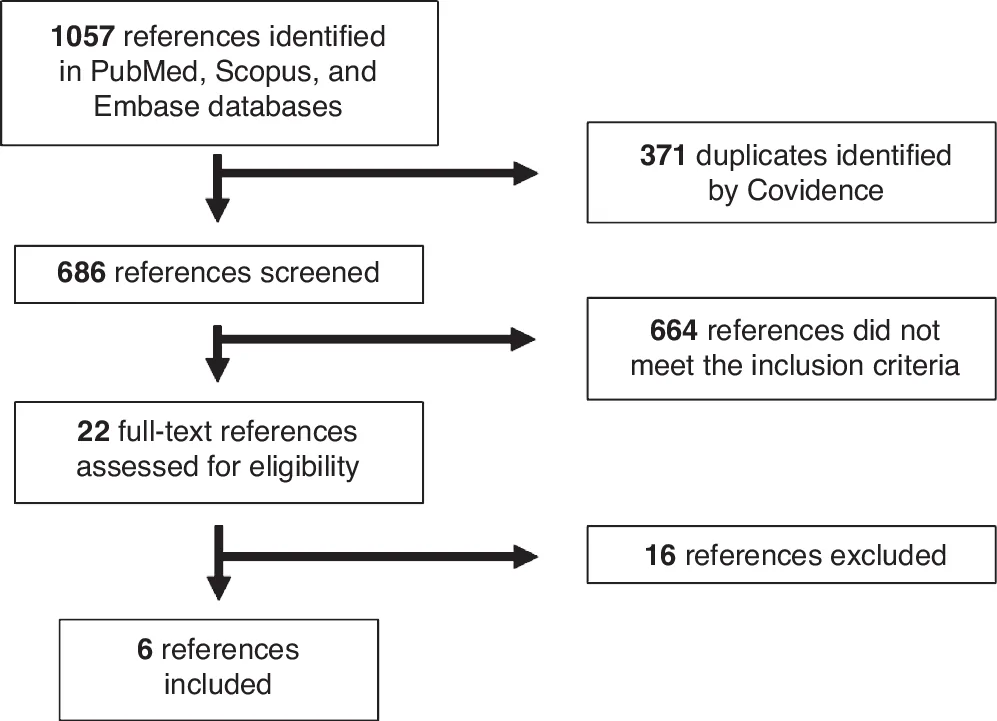
Diagram of manuscript identification and screening.

We included randomized controlled trials (RCTs), clinical trials, guidelines, observational studies, systematic reviews, and scoping reviews. We restricted articles to those published from 2005 to 2026, peer-reviewed, written in English, and conducted in humans ages 0–18 years. We excluded articles that were explicitly about air filtration to prevent coronavirus disease 2019 (COVID-19) infection, though we chose to include articles that mentioned COVID-19 in the context of indoor air pollution more broadly. In our second and third round of screening, references were most commonly excluded because they did not include an intervention or because they investigated pulmonary outcomes such as asthma and respiratory infections. The two literature searches identified 1057 references of interest. References were uploaded into Endnote and Covidence, a web-based software service to streamline production of literature reviews (Veritas Health Innovation, Melbourne, Australia, 2025), for further screening by two reviewers. Covidence identified 371 duplicate references that were eliminated. Two reviewers performed title/abstract screening and discussed any conflicts together to come to a mutual agreement. Supplemental Table S2 outlines all inclusion and exclusion criteria. Inclusion criteria included the study designs and study population noted above, as well as the presence of one of the exposures and interventions of interest (e.g., PM2.5 and other particulates, HEPA filtration) and a comparison group with no or standard air filtration. Additionally, the studies had to include a neurobehavioral outcome and air quality measures. Studies were excluded if they included no intervention or a pharmacological intervention, if they solely measured exposure to ozone or other atmospheric gases or did not contain any air pollution measurements, and if the outcome was not neurobehavioral, such as respiratory outcomes (e.g., asthma, pneumonia). Twenty-two full-text articles were assessed for eligibility, with 16 studies excluded due to an ineligible study design (4), having no intervention (5), not measuring one of the exposures of interest (4), having no neurodevelopmental outcome of interest (1), and only describing a protocol (1). Figure 1 includes a reference selection diagram.

## Results

Upon completion of manuscript screening, we identified six relevant manuscripts: four manuscripts from the Ulaanbaatar Gestation and Air Pollution Research (UGAAR) RCT and two additional manuscripts (Table 1). The UGAAR trial was conducted in highly polluted Ulaanbaatar, the capital of Mongolia.^45^ The trial enrolled 540 women at ≤18 weeks gestation and randomly assigned them to receive 1–2 HEPA cleaners or no HEPA cleaners in their home during the rest of their pregnancy. The HEPA filter intervention was associated with a 29% reduction (95% confidence interval (CI): 21%, 37%) in mean indoor PM_2.5_. In Enkhbat et al.^45^, the Behavior Assessment System for Children (BASC-3) was administered to caregivers at 2-year and 4-year follow-up visits. In the primary intention-to-treat analysis, there was no significant effect of the HEPA intervention on any of the four BASC-3 composite scores assessing internalizing problems, externalizing problems, behavioral symptoms, and adaptive skills. However, in secondary observational analyses, higher indoor PM_2.5_ concentration during the first trimester was associated with higher mean *T*-scores for internalizing and externalizing problems, and lower mean *T*-scores for the adaptive skills at age 4 years. Additionally, higher indoor PM_2.5_ concentration during the third trimester was associated with higher mean *T*-scores for internalizing problems at age 4 years, and higher indoor PM_2.5_ concentration throughout pregnancy was associated with higher mean Behavioral Symptoms Index *T*-scores at age 4 years. Additional outcomes assessed in the UGAAR trial included full-scale intelligence quotient (FSIQ), measured via Wechsler Preschool and Primary Scale of Intelligence (WPPSI-IV),^46^ and effect modifiers of the association between the intervention and FSIQ,^46^ as well as social responsiveness, measured with the Social Responsiveness Scale-2 (SRS-2).^47^ In the primary intention-to-treat analysis, children in the HEPA intervention group had a 2.5-point higher mean FSIQ, though the confidence interval crossed the null (95% confidence interval: -0.4, 5.4 IQ points). In complete case analysis, children in the HEPA intervention group had a 3.2-point higher mean FSIQ at age 4 years. A follow-up study of 242 mother-child dyads found significant interaction between maternal hair cortisol during pregnancy and HEPA intervention in the association between HEPA intervention and FSIQ at age 4 years. This suggests the intervention may be more beneficial in children whose mothers experienced higher levels of cortisol—the major stress hormone – during pregnancy. Furthermore, secondary analyses noted the largest between-group differences in the effect of the HEPA intervention by maternal prenatal vitamin use, maternal education level, maternal hair dehydroepiandrosterone (DHEA), and maternal perceived stress score (PSS-4) during pregnancy. Lastly, in Enkhbat et al.^47^, HEPA intervention was not significantly associated with lower mean SRS-2 *T*-scores in primary analysis, but an interquartile range increase in indoor PM_2.5_ concentration throughout pregnancy was associated with a 1.8-unit increase in mean SRS-2 *T*-score.

**Table 1:** Included studies, assessment of effect on outcome.

Study	Detail	Study characteristics	Intervention	Outcome measure(s)	Result	Statistically significant?
UGAAR Prenatal intervention	Enkhbat, et al.^45^	Single-blind parallel-group randomized controlled trial *N* = 478 children (233 control, 245 intervention) *N* = 242 mother-child dyads	Pregnant women enrolled at 5–19 weeks gestation were randomized to receive HEPA cleaner in their home throughout pregnancy	Mean indoor PM_2.5_ concentration during pregnancy	Reduced by 29%	Yes (95% CI: 21%, 37%)
				BASC-3 internalizing problems score at ages 2 and 4 years	No significant effect of intervention on scores in ITT analysis Increased indoor PM2.5 concentration during 1st and 3rd trimester associated with higher mean *T*-score at age 4 years	No (primary analysis) Yes (secondary analyses)
				BASC-3 externalizing problems score at ages 2 and 4 years	No significant effect of intervention on scores in ITT analysis Increased indoor PM2.5 concentration during 1st trimester associated with higher mean *T*-score at age 4 years	No (primary analysis) Yes (secondary analyses)
				BASC-3 adaptive skills score at ages 2 and 4 years	No significant effect of intervention on scores in ITT analysis Increased indoor PM2.5 concentration during 1st trimester associated with lower mean *T*-score at age 4 years	No (primary analysis) Yes (secondary analyses)
				BASC-3 Behavioral Symptoms Index at ages 2 and 4 years	No significant effect of intervention on scores in ITT analysis Increased indoor PM2.5 concentration during pregnancy associated with higher mean *T*-score at age 4 years	No (primary analysis) Yes (secondary analyses)
	Ulziikhuu, et al.^46^			Full-scale intelligence quotient (FSIQ) from Wechsler Preschool and Primary Scale of Intelligence (WPPSI-IV) at age 4 years	In ITT analysis, children in the intervention group had a 2.5-point higher mean FSIQ	No (95% CI: −0.4, 5.4)
	Enkhbat, et al.^47^			Social Responsiveness Scale-2 (SRS-2) *T*-score at age 4 years	Mean SRS-2 total *T*-score was 0.5 (95% CI: −2.5 to 1.5) units lower in intervention group IQR increase in indoor PM_2.5_ concentration over full pregnancy associated with 1.8-unit increase in mean SRS-2 *T*-score.	No (primary analysis) Yes (secondary analysis)
	Ulziikhuu, et al.^65^			Effect modifiers of the association between intervention group and mean FSIQ via WPPSI-IV at age 4 years by various maternal and sociodemographic factors	Significant interaction of maternal hair cortisol and intervention Largest between-group differences in effect of the intervention by maternal vitamin use, maternal education, maternal hair DHEA, and perceived stress scores (PSS-4).	Yes (interaction *p*-value maternal hair cortisol = 0.02) No (secondary analyses)
Aliso Canyon gas leak	Gilraine^48^	Observational study following gas leak that occurred 2015–2016 and affected 18 schools	Air filtration installed in classrooms located within 5 miles of Aliso Canyon gas leak	Classroom air quality testing after filter placement Standardized math and English test scores after filter placement	Significant reduction in ethylbenzene and xylenes Significant increase in mean mathematics scores by 0.1–0.2 standard deviations (SD) but not English test scores	Yes (alpha = 0.01) Yes (alpha = 0.05)
North Carolina school air purification upgrades	Perisco and Fuller^49^	Observational study utilizing data from all North Carolina public schools	HVAC fixes or upgrades performed 2020–2023	Student outcomes: standardized test scores, absences, and grades	HVAC updates associated with 3.3% SD higher test scores, 2.7% fewer absences, 3.7% fewer chronic absences, and a 10% increase in letter grade.	Yes (alpha = 0.01)

*BASC-3* Behavior Assessment System for Children, *HEPA* High Efficiency Particulate Air, *TT* intention-to-treat, *PM* particulate matter, *HVAC* heating, ventilation, and air conditioning, *ESSER* Elementary and Secondary School Emergency Relief.

The remaining two manuscripts utilized natural experiments to assess academic outcomes. In one study by Gilraine, he leveraged corrective actions that occurred during the 2015 Aliso Canyon methane gas leak near Los Angeles, California.^48^ Air filters were installed in classrooms located within a five-mile radius of the gas leak. Gilraine analyzed classroom air quality test results and standardized test scores at schools within a five-mile radius and at schools one mile beyond the boundary that did not receive air filters. In classrooms where air filters were installed, children had significantly higher mean mathematics scores (0.1–0.2 standard deviations). There was also a significant reduction in ethylbenzene and xylene concentrations, VOCs associated specifically with the gas leak. There was no significant difference in mean English test scores between the groups, though the estimate was positive in direction for schools within the five-mile radius of the gas leak.

Persico and Fuller evaluated student outcomes after heating, ventilation, and air conditioning (HVAC) upgrades in North Carolina public schools from 2020–2023.^49^ They used public school data, Air Quality Index data from the US EPA, and school-level data from Public School Building Capital Fund reports and the Elementary and Secondary School Emergency Relief (ESSER) program to compare students in schools with and without HVAC upgrades. HVAC upgrades were associated with higher standardized test scores and letter grades, along with fewer absences and decreased chronic absenteeism.

The UGAAR trial was the only relevant RCT we identified in our two-person screening process. The rigorous study design and rich data collected are clear strengths of this study. However, the UGAAR trial was powered to detect birth weight outcomes and may be underpowered to detect significant differences in cognitive and neurobehavioral outcomes. Additionally, study team members were blinded to study arm randomization, but study participants were not blinded to the intervention (HEPA filter placement). The authors note this could have contributed to greater loss to follow up in the control group. This may also contribute to bias in caregiver-reported assessments such as the BASC-3 and SRS-2. Furthermore, as others have noted,^50^ exposure measurements were not obtained prior to the HEPA intervention. Thus, one cannot solely attribute the observed reduction in PM_2.5_ and associate better neurobehavioral outcomes to the intervention. Lastly, this study was performed in Ulaanbaatar, Mongolia. The majority of PM_2.5_ in Ulaanbaatar comes from coal home heating stoves, and it is one of the most polluted areas in the world.^51^ Future studies could address these limitations and replicate the investigation in areas of the world with more moderate air pollution levels. The remaining studies leveraged natural experiments that occurred due to a methane gas leak^48^ and due to HVAC upgrades performed with ESSER funding.^49^ These studies include robust sample sizes but lack finer detail in the air pollution exposure and outcomes measured. Only Gilraine’s study reported air pollutant concentrations after the air filter intervention, whereas in Persico and Fuller’s study it was assumed that HVAC upgrades decreased air pollutant concentrations.

## Discussion

In this narrative review, we completed multiple rounds of literature search and screening to identify studies of interest. Ultimately, we identified six manuscripts of interest describing three studies of indoor air quality interventions and neurobehavioral outcomes. These studies provide valuable insights into the potential benefits of interventions to reduce indoor air pollution for neurobehavioral outcomes. Overall, our review found a striking lack of studies on indoor air interventions and neurobehavioral outcomes despite epidemiologic evidence of harmful effects of air pollution on neurobehavioral outcomes. Given that interventional studies show promising benefits of indoor air pollution interventions for respiratory health,^26,27^ this is a notable gap in the research. Epidemiologic and animal studies provide ample evidence that reducing air pollution would likely benefit children’s neurobehavioral outcomes. For example, children in Mexico City were exposed to PM_2.5_ concentrations significantly higher (25 μg/m^3^) than annual US air quality standards (9 μg/m^3^)^52^ and displayed impaired cognitive performance and prefrontal white matter hyperintense lesions on brain magnetic resonance imaging (MRI) when compared to children living in Polotitlán, a nearby city with air pollutant concentrations below US air quality standards.^18^ In the Upstate KIDS prospective birth cohort, indoor exposure to unclean cooking fuels, which produce a mixture of indoor air pollutants including PM, NO_2_, CO, and PAHs, was associated with increased odds of developmental delay, assessed via the Ages and Stages Questionnaire.^53^ Additionally, short-term exposures to air pollution are associated with acute mental health effects in children. For example, acute increases in PM_2.5_ were associated with increased psychiatric emergency department visits 1 and 2 days later.^54^ In a developmental mouse model of ambient particulate matter exposure, mice displayed impaired short-term memory and learning, motor function in behavioral tasks, as well as sustained glial activation and glutamate-dopamine neurotransmitter imbalances.^55^

Children are uniquely susceptible to the adverse effects of indoor air pollution due to their significant time spent indoors, higher minute ventilation, and developing immune systems. In addition, neurodevelopment is a carefully orchestrated process particularly vulnerable to external perturbations.^10,20^ Thus, the neurobehavioral impacts of indoor air pollution may be especially influential in children. Interventions designed to reduce and/or mitigate indoor air pollution could potentially reap significant benefits for neurobehavioral endpoints across the lifespan.

While RCTs can be difficult to conduct for environmental health hazards, there would ideally be more work done in this area. This is an area where other quasi-experimental designs, such as target trial emulation, could be useful. This study design can reduce bias in observational data by selecting only data that meet strict operational definitions of a hypothetical RCT.^56^ There were no case-control or cross-sectional studies identified in our screening process, for example. Furthermore, the current studies focus on prenatal and school-based exposures. Given the neurobehavioral results noted in the UGAAR trial’s secondary analyses, it would be valuable to incorporate repeated timepoints between gestation and kindergarten to clarify windows of susceptibility to the exposure and optimal timeframes for the intervention. Additional studies suggest that potential interventions could be relatively inexpensive and accessible. One study compared lower-cost air filter options, such as Corsi-Rosenthal box filters, with HEPA filtration.^57^ Beyond air filtration, another avenue for intervention is nutrition, as dietary components such as fish consumption have been associated with neuroprotective effects in offspring of pregnant women exposed to PM_2.5._^58^

Potential interventions should be directed towards reducing air pollution exposure in the places where children spend most of their time indoors: at home, at school, and in childcare settings. Since children spend about one-third of their day at school, schools are a significant setting for both exposure to air pollution and promoting exposure to healthy air.^59^ For newly constructed schools, there is existing guidance on school siting from the U.S. EPA,^60^ but many schools are already impacted by exposure to high ambient air pollution^61,62^ and will require interventions to improve their indoor air quality.

Beyond air filtration strategies, policies for indoor air quality standards may also be a promising avenue. There are currently no policies set forth by the U.S. EPA for residential or school-based indoor air quality standards. The U.S. Clean Air Act regulates six outdoor air pollutants based on their known health impacts, including particulate matter.^63^ However, 59.3% of U.S. children lived in a county where air pollutant concentrations exceeded at least one air quality standard in 2021.^64^

Strengths of this review include a broad search strategy and screening of over 1000 references, as well as our focus on identifying potential interventions. Limitations of our review include the large number of references that did not meet our inclusion criteria. The relatively small number of references included makes substantial synthesis of information difficult. There is also potential for bias in this narrative review due to the limited number of studies, particularly because two of the studies were ecological. Additionally, the UGAAR randomized-controlled trial was originally designed to measure fetal growth as the primary outcome of interest (ClinicalTrials.gov: NCT01741051).

As evidence examining the associations between indoor air pollution and neurobehavioral outcomes grows, we hope that additional information on appropriate interventions will also emerge. Early findings from a small body of literature on interventions for indoor air quality and neurobehavioral outcomes suggest that early-life air filtration may be beneficial for neurobehavioral outcomes such as cognition, behavioral symptoms, social skills, and academic performance in certain groups of school-age children. Further study of potential interventions is warranted, as this information is critical to develop policies and public health programs that mitigate the adverse effects of indoor air pollution on neurobehavioral outcomes in children.

## Supplementary information

Supplementary information
