# Supplementary material for: A review of indoor air quality interventions and child neurobehavioral outcomes
Source: Pediatr Res. 2026 Aug 27;100(4):1598–604. doi: 10.1038/s41390-026-05401-w (PMC13759306; doi:10.1038/s41390-026-05401-w)
Supplement: Supplementary file 1 [file 41390_2026_5401_MOESM1_ESM.pdf]

Supplemental Table S1: Search terms used for each database

Database	Search string
**Initial literature search 9/22/2025**	
PubMed 1	("air quality"[Title/Abstract] OR "particle pollution"[Title/Abstract] OR "indoor air quality"[Title/Abstract] OR "indoor air pollution"[Title/Abstract]) AND (Neurodevelopment[Title/Abstract] OR cognition[Title/Abstract] OR "mental health"[Title/Abstract]) Filters: in the last 10 years, English, Humans, Child: birth-18 years
PubMed 2	("air filtration"[Title/Abstract] OR "air filter*"[Title/Abstract] OR "indoor air filtration"[Title/Abstract] OR "HEPA filtration"[Title/Abstract] OR "HEPA filter*"[Title/Abstract] OR "box fan filter"[Title/Abstract]) AND (Neurodevelopment[Title/Abstract] OR cognition[Title/Abstract] OR "mental health"[Title/Abstract]) Filters: in the last 10 years, English, Humans
Scopus	(TITLE-ABS-KEY ("air filtration" OR "air filter*" OR "indoor air filtration" OR HEPA OR "HEPA filtration" OR "HEPA filter*" OR "box fan filter" OR "Corsi-Rosenthal box") AND TITLE-ABS-KEY (Neurodevelopment OR cognition OR "mental health")) AND PUBYEAR > 2014 AND (LIMIT-TO (DOCTYPE , "ar")) AND (LIMIT-TO (LANGUAGE , "English"))
Embase	('air filtration':ti,ab,kw OR 'air filter*':ti,ab,kw OR 'indoor air filtration':ti,ab,kw OR hepa:ti,ab,kw OR 'hepa filtration':ti,ab,kw OR 'hepa filter*':ti,ab,kw OR 'box fan filter':ti,ab,kw OR 'corsi-rosenthal box':ti,ab,kw) AND (neurodevelopment:ti,ab,kw OR cognition:ti,ab,kw OR 'mental health':ti,ab,kw) AND [2015-2025]/py
Google Scholar	“air quality” OR “particle pollution” OR “indoor air quality” OR “indoor air pollution” AND “air filtration” OR “air filter*” OR “indoor air filtration” OR “HEPA filtration” OR “HEPA filter*” OR “box fan filter” AND Neurodevelopment OR cognition OR “mental health” AND child or pediatric<br>2015-2025<br>Exclude citations
**Literature search 9/30/2025**	
PubMed 1	("air quality"[Title/Abstract] OR "particle pollution"[Title/Abstract] OR "indoor air quality"[Title/Abstract] OR "indoor air pollution"[Title/Abstract]) AND ("health condition"[Title/Abstract] OR condition[Title/Abstract] OR disorder[Title/Abstract] OR outcome[Title/Abstract]) Filters: in the last 10 years, English, Humans, Child: birth-18 years
PubMed 2	("air filtration"[Title/Abstract] OR "air filtering"[Title/Abstract] OR "indoor air filtration"[Title/Abstract] OR HEPA[Title/Abstract] OR "HEPA filtration"[Title/Abstract] OR "HEPA filter"[Title/Abstract] OR

	"box fan filter"[Title/Abstract] OR "Corsi-Rosenthal box"[Title/Abstract]) AND ("health condition"[Title/Abstract] OR condition[Title/Abstract] OR disorder[Title/Abstract] OR outcome[Title/Abstract]) Filters: in the last 10 years, English, Humans, Child: birth-18 years
Scopus 1	(TITLE-ABS-KEY ("air quality" OR "particle pollution" OR "indoor air quality" OR "indoor air pollution") AND TITLE-ABS-KEY ("health condition" OR condition OR disorder OR outcome) AND TITLE-ABS-KEY (pediatric OR child)) AND PUBYEAR > 2014 AND (LIMIT-TO (EXACTKEYWORD , "Human") OR LIMIT-TO (EXACTKEYWORD , "Humans") OR LIMIT-TO (EXACTKEYWORD , "Child")) AND (LIMIT-TO (DOCTYPE , "ar")) AND (LIMIT-TO (LANGUAGE , "English"))
Embase1	('air quality':ab,ti OR 'particle pollution':ab,ti OR 'indoor air quality':ab,ti OR 'indoor air pollution':ab,ti) AND ('health condition':ab,ti OR condition:ab,ti OR disorder:ab,ti OR outcome:ab,ti) AND (pediatric:ab,ti OR child:ab,ti) AND ([adolescent]/lim OR [child]/lim)
Embase 2	('air filtration':ab,ti OR 'air filtering':ab,ti OR 'indoor air filtration':ab,ti OR hepa:ab,ti OR 'hepa filtration':ab,ti OR 'hepa filter':ab,ti OR 'box fan filter':ab,ti OR 'corsi-rosenthal box':ab,ti) AND ('health condition':ab,ti OR condition:ab,ti OR disorder:ab,ti OR outcome:ab,ti) AND (pediatric:ab,ti OR child:ab,ti) AND [english]/lim AND ([child]/lim OR [adolescent]/lim)
**Literature search completed 3/10/2026**	
PubMed 1	("Air Pollution, Indoor"[Mesh] OR "Particulate Matter"[Mesh] OR "air quality"[tiab] OR "indoor air quality"[tiab] OR "indoor air pollution"[tiab] OR "particle pollution"[tiab] OR "particulate pollution"[tiab]) AND ("Air Filters"[Mesh] OR "Ventilation"[Mesh] OR "air filtration"[tiab] OR "air filtering"[tiab] OR "indoor air filtration"[tiab] OR "HEPA"[tiab] OR "HEPA filter*"[tiab] OR "HEPA filtration"[tiab] OR "portable air cleaner*"[tiab] OR "room air cleaner*"[tiab] OR "box fan filter*"[tiab] OR "Corsi-Rosenthal"[tiab]) Filters: English, Humans, Child: birth-18 years, from 2005 - 2026
PubMed 2	(("Air Pollution, Indoor"[Mesh] OR "indoor air quality"[tiab] OR "indoor air pollution"[tiab]) AND ("Air Filters"[Mesh] OR "Ventilation"[Mesh] OR "air filtration"[tiab] OR "air filtering"[tiab] OR "indoor air filtration"[tiab] OR "HEPA"[tiab] OR "HEPA filter*"[tiab] OR "HEPA filtration"[tiab] OR "portable air cleaner*"[tiab] OR "room air cleaner*"[tiab] OR "box fan filter*"[tiab] OR "Corsi-Rosenthal"[tiab])) AND (disease OR disorder OR illness OR syndrome OR disability OR outcome OR chronic illness OR chronic disease) Filters: English, Humans, Child: birth-18 years, from 2005 - 2026
Scopus 1	(TITLE-ABS-KEY ("indoor air quality" OR "indoor air pollution") AND TITLE-ABS-KEY ("Air Filters" OR "Ventilation" OR "air

	filtration" OR "air filtering" OR "indoor air filtration" OR "HEPA" OR "HEPA filter*" OR "HEPA filtration" OR "portable air cleaner*" OR "room air cleaner*" OR "box fan filter*" OR "Corsi-Rosenthal") AND TITLE-ABS-KEY (child OR children OR pediatric)) AND PUBYEAR > 2005 AND (LIMIT-TO (DOCTYPE , "ar")) AND (LIMIT-TO (LANGUAGE , "English")) AND (LIMIT-TO (EXACTKEYWORD , "Human") OR LIMIT-TO (EXACTKEYWORD , "Child") OR LIMIT-TO (EXACTKEYWORD , "Humans"))
Scopus 2	(TITLE-ABS-KEY ("indoor air quality" OR "indoor air pollution") AND TITLE-ABS-KEY ("Air Filters" OR "Ventilation" OR "air filtration" OR "air filtering" OR "indoor air filtration" OR "HEPA" OR "HEPA filter*" OR "HEPA filtration" OR "portable air cleaner*" OR "room air cleaner*" OR "box fan filter*" OR "Corsi-Rosenthal") AND TITLE-ABS-KEY (disease OR disorder OR illness OR syndrome OR disability OR outcome OR chronic illness OR chronic disease) AND TITLE-ABS-KEY (child OR children OR pediatric OR adolescent)) AND PUBYEAR > 2005 AND (LIMIT-TO (DOCTYPE , "ar")) AND (LIMIT-TO (LANGUAGE , "English")) AND (LIMIT-TO (EXACTKEYWORD , "Human") OR LIMIT-TO (EXACTKEYWORD , "Child") OR LIMIT-TO (EXACTKEYWORD , "Humans") OR LIMIT-TO (EXACTKEYWORD , "Children"))
Embase 1	('indoor air quality':ti,ab,kw OR 'indoor air pollution':ti,ab,kw) AND ('air filters':ti,ab,kw OR 'ventilation':ti,ab,kw OR 'air filtration':ti,ab,kw OR 'air filtering':ti,ab,kw OR 'indoor air filtration':ti,ab,kw OR 'hepa':ti,ab,kw OR 'hepa filter*':ti,ab,kw OR 'hepa filtration':ti,ab,kw OR 'portable air cleaner*':ti,ab,kw OR 'room air cleaner*':ti,ab,kw OR 'box fan filter*':ti,ab,kw OR 'corsi-rosenthal':ti,ab,kw) AND (child:ti,ab,kw OR children:ti,ab,kw OR pediatric:ti,ab,kw OR adolescent:ti,ab,kw) AND [2005-2026]/py
Embase 2	('indoor air quality':ti,ab,kw OR 'indoor air pollution':ti,ab,kw) AND ('air filters':ti,ab,kw OR 'ventilation':ti,ab,kw OR 'air filtration':ti,ab,kw OR 'air filtering':ti,ab,kw OR 'indoor air filtration':ti,ab,kw OR 'hepa':ti,ab,kw OR 'hepa filter*':ti,ab,kw OR 'hepa filtration':ti,ab,kw OR 'portable air cleaner*':ti,ab,kw OR 'room air cleaner*':ti,ab,kw OR 'box fan filter*':ti,ab,kw OR 'corsi-rosenthal':ti,ab,kw) AND (disease:ti,ab,kw OR disorder:ti,ab,kw OR illness:ti,ab,kw OR syndrome:ti,ab,kw OR disability:ti,ab,kw OR outcome:ti,ab,kw OR 'chronic illness':ti,ab,kw OR 'chronic disease':ti,ab,kw) AND (child:ti,ab,kw OR children:ti,ab,kw OR pediatric:ti,ab,kw OR adolescent:ti,ab,kw) AND [2005-2026]/py AND [english]/lim

Supplemental Table S2: Study inclusion and exclusion criteria

	**Inclusion Criteria**	**Exclusion Criteria**
**Study Characteristics**	• RCTs<br>• Observational studies (natural experiments)<br>• Case control<br>• Cross sectional	• Commentaries<br>• Reviews<br>• Studies that did not include an intervention or natural experiment
**Study population**	• Children (<19 years)	• Non-human studies<br>• Adult studies
**Intervention/Exposure**	• Air filtration (including box fan filters)<br>• Air conditioning<br>• HEPA filtration<br>• PM2.5 and other particulates<br>• Wildfire smoke<br>• PPE<br>• Objective air pollution measurements (outdoor or indoor)	• Pharmacological intervention<br>• Ozone exposure<br>• Other atmospheric gases (VOCs, CO2, CO, NO2)
**Comparator/Context**	• Standard air conditioning/HVAC without filtration<br>• No air filtration	• No air pollution measurements
**Outcome**	• Neurobehavioral/developmental<br>• Air quality measures<br>• ADHD<br>• Autism<br>• ASD<br>• Cognitive<br>• IQ<br>• Speech, lanugage, or communication delay	• Outcomes that do not measure health or air quality<br>• Non-neurological/neurobehavioral outcomes<br>• Asthma<br>• Eczema<br>• Pneumonia
